# Intratumoral hemorrhage in vestibular schwannomas post-radiosurgery: a systematic review

**DOI:** 10.1186/s41016-026-00439-5

**Published:** 2026-06-26

**Authors:** Areeba Fareed, Kashif Qureshi, Solay Farhat, Bipin Chaurasia, Abdulrahmon Moradeyo, Laura Ghanem, Rayyan Vaid, Syed Muhammad Muneeb Akhtar, Issa Awaida, Abed AlRazzak Kerhani, Kivanc Yangi

**Affiliations:** 1 Department of Medicine, Karachi Medical and Dental College, Karachi, Pakistan; 2 Department of Neurosurgery, Yale School of Medicine, New Haven, CT USA; 3 Faculty of Medical Sciences, Lebanese University, Beirut, Lebanon; 4 Department of Neurosurgery, Neurosurgery Clinic, Birgunj, Nepal; 5 Department of Medicine and Surgery, Ladoke Akintola University of Technology, Ogbomosh, Nigeria; 6 Department of Medicine, Dow University of Health Sciences, Karachi, Pakistan; 7 Faculty of Medicine, University of Balamand, Balamand, Lebanon; 8 Department of Neurosurgery, Barrow Neurological Institute, Phoenix, AZ USA

**Keywords:** Vestibular schwannomas (VS), Acoustic neuromas, Stereotactic radiosurgery (SRS), Intratumoral Hemorrhage (ITH)

## Abstract

**Background:**

Vestibular schwannomas (VS) are benign tumors affecting the vestibulocochlear nerve. They cause symptoms like hearing loss and vertigo. Treatment options include surgery and stereotactic radiosurgery (SRS). However, SRS can lead to rare complications like intratumoral hemorrhage (ITH). This review aims to explore ITH post-SRS, including its incidence and risk factors, to improve patient care.

**Methods:**

This systematic review, conducted in accordance with PRISMA 2020 guidelines, examines intratumoral hemorrhage (ITH) post-radiosurgery for vestibular schwannomas. Searches encompassed PubMed, Google Scholar, the Cochrane Library, Embase, Web of Science, and Scopus up to January 2024. Inclusion criteria required documented vestibular schwannoma with confirmed ITH occurring after SRS. Data extraction involved study and participant characteristics, with discrepancies resolved by discussion. Given clinical and methodological heterogeneity, quantitative pooling was not performed; results are presented as a qualitative narrative synthesis.

**Results:**

Eleven studies comprising 47 patients met inclusion criteria. Patients ranged from 25 to 79 years of age, with clustering in the sixth decade. A subset of patients had identifiable bleeding risk factors, most commonly hypertension and anticoagulant use. Symptom profiles were heterogeneous; trigeminal neuropathy and disequilibrium were the most frequently reported presentations. Management ranged from observation to surgical resection. On descriptive analysis, approximately half of patients demonstrated outcomes classified as favorable by the reporting authors; mortality was reported in 2 of 47 patients (4.35%). These figures must be interpreted with caution given the small total sample, predominance of case reports, and absence of standardized outcome definitions across included studies.

**Conclusion:**

In conclusion, managing intratumoral hemorrhage following radiosurgery for vestibular schwannomas remains a complex clinical challenge. This review highlights varied patient outcomes, emphasizing the importance of personalized treatment approaches. Tailoring treatment decisions to individual factors such as tumor characteristics and patient preferences is crucial for optimizing outcomes and minimizing complications, underscoring the need for close collaboration among specialists in clinical practice.

**Systematic review registration:**

PROSPERO CRD42024511320.

**Graphical Abstract:**

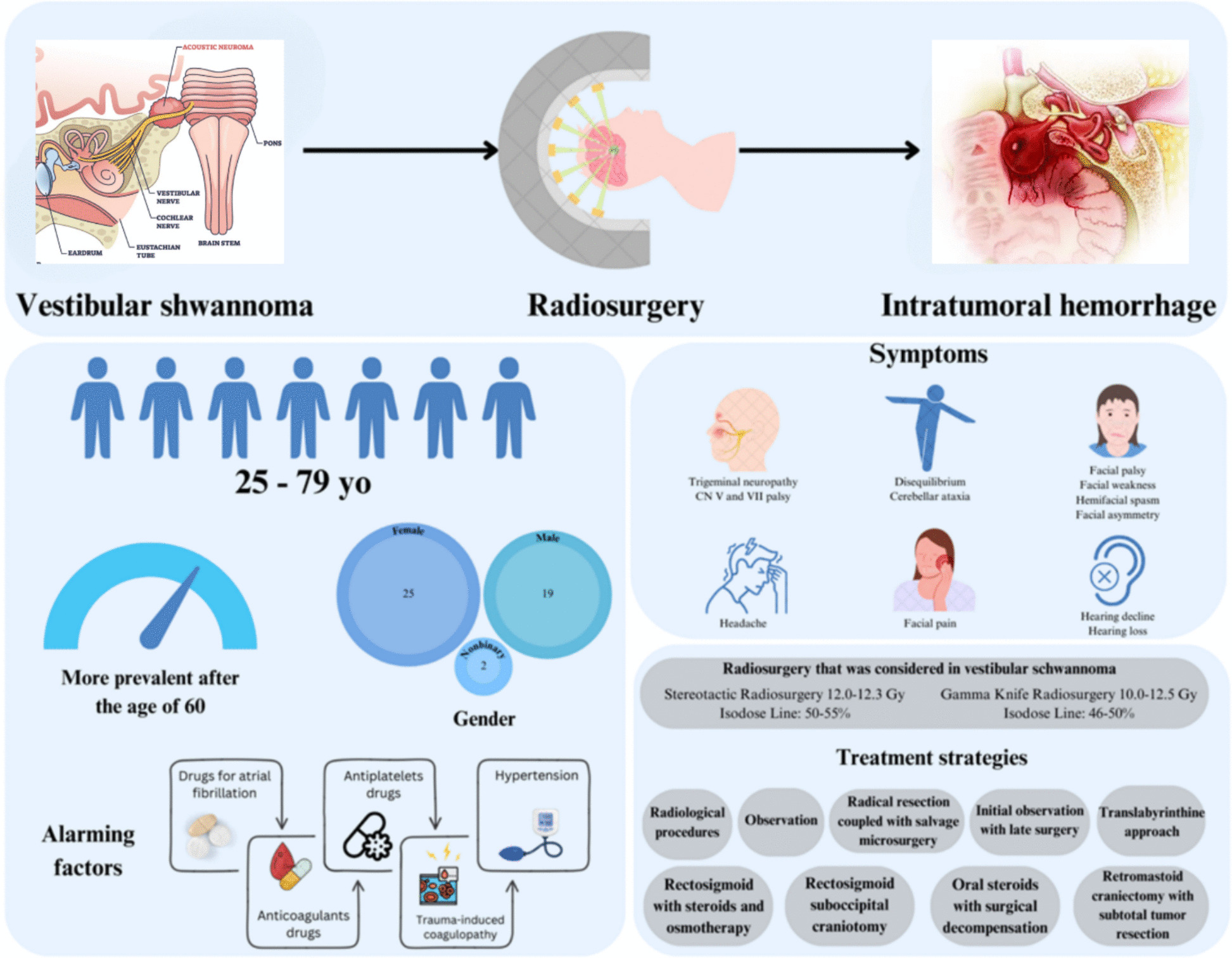

**Supplementary Information:**

The online version contains supplementary material available at 10.1186/s41016-026-00439-5.

## Background

Vestibular schwannomas (VS), also known as acoustic neuromas, are benign tumors that arise from the vestibulocochlear nerve, impacting auditory and vestibular functions in the inner ear. With an annual incidence of 10.4 cases per million people, vestibular schwannomas make up roughly 8% of all intracranial tumors. They are mostly found at the cerebellopontine angle and are typically unilateral and sporadic in nature [1, 2]. While they usually affect one ear, bilateral cases are associated with neurofibromatosis type 2 (NF2), a hereditary disorder caused by mutations in the NF2 gene. These mutations lead to uncontrolled cell growth, including the development of VS; symptoms vary based on tumor size, location, and growth rate [3, 4]. They commonly include unilateral hearing loss, tinnitus, vertigo, and facial weakness as the tumor grows and affects nearby nerves [4]. Diagnosis involves a thorough evaluation, including audiometry, vestibular function tests, and MRI imaging with contrast enhancement to visualize the tumor's characteristics [5]. Treatment options depend on factors like tumor size, growth rate, and patient preferences. They include active surveillance, microsurgery, and fractionated radiotherapy [6, 7].

Stereotactic radiosurgery (SRS), particularly in the context of VS treatment, is a method utilized for managing smaller tumors [8]. This technique employs focused radiation or energy beams to eliminate tumor cells, with the aim of halting the growth of the VS without resorting to surgical removal. It is especially advantageous when traditional surgery is not viable due to health concerns or advanced age. By precisely targeting the tumor, such as with Gamma Knife® surgery, this approach strives to deactivate the tumor while safeguarding the function of cranial nerves and minimizing harm to vital nerves responsible for hearing, balance, facial movement, and sensation. Gamma Knife® surgery delivers radiation with pinpoint accuracy, thereby mitigating the risks linked with traditional open surgery [9]. Indeed, studies have noted that stereotactic radiosurgery is an efficient method for handling vestibular schwannomas, demonstrating substantial success in tumor control with few complications [8, 10]. Additionally, for substantial VS, fractionated stereotactic radiotherapy (FSRT) has demonstrated effectiveness, effectively controlling local tumor growth while causing minimal harm to nearby structures such as the trigeminal and facial nerves [11]. Employing FSRT for sizable VS offers a non-surgical alternative for patients who may not be candidates for surgery or single-dose SRS.

Although stereotactic radiosurgery offers higher rates of tumor control and low risks of complications like cerebrospinal fluid leakage or facial palsy [8, 12], though rare but clinically significant complications including intratumoral hemorrhage have been documented. This review defines intratumoral hemorrhage (ITH) in vestibular schwannomas using both radiological and clinical criteria. Radiologically, ITH is identified using magnetic resonance imaging (MRI), which produces hypointense or hyperintense signals on T1- and T2-weighted images, indicating the presence of blood within the tumor. Clinically, ITH presents with symptoms such as sudden hearing loss, facial nerve deficits, vertigo, or headache, which typically correspond with imaging findings. This comprehensive review delves into the existing literature on ITH in vestibular schwannoma post-radiosurgery. The goal is to establish the overall incidence, identify associated risk factors, and determine expected complication rates, thereby equipping clinicians with vital information to better counsel their patients.

## Methods

**Fig. 1:**
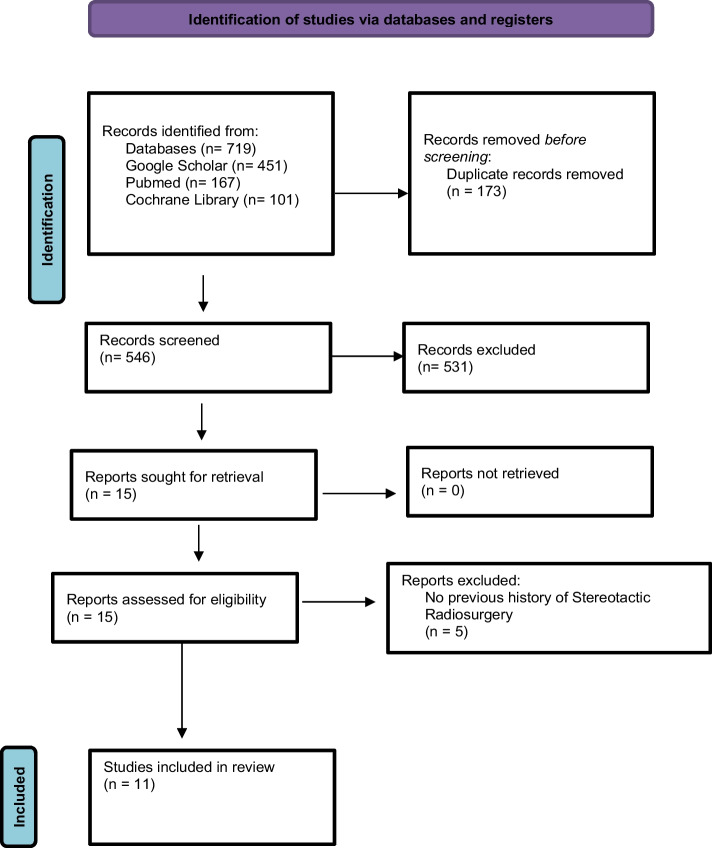
PRISMA Flow Diagram of the Literature Search Process

This systematic review was conducted in accordance with the Preferred Reporting Items for Systematic Reviews and Meta-Analyses (PRISMA) 2020 guidelines [13], as shown in Fig. 1. Our protocol was registered with PROSPERO, The International Prospective Register of Systematic Reviews with registration no. CRD42024511320.

### Data sources and search strategies

An electronic search of PubMed, Google Scholar, the Cochrane Library, Embase, Web of Science, and Scopus was conducted. The search extended to all available English-language articles till January 2024 using a search strategy created using Boolean operators. We conducted an extensive search using following terms “intratumoral hemorrhage” “acoustic neuroma” OR “vestibular schwannoma” “Stereotactic radiosurgery” OR “gamma knife radiosurgery”. Detailed searched strategy is provided in Supplementary Table S1.

### Inclusion and exclusion criteria

Our research focused exclusively on papers presenting distinct clinical characteristics indicative of intratumoral hemorrhage (ITH) following stereotactic radiosurgery (SRS) or Gamma Knife Surgery (GKS). We included only cases of vestibular schwannoma in which intratumoral hemorrhaging occurred after stereotactic radiosurgery (SRS), ensuring that SRS was not the therapy for the hemorrhage itself. Studies that reported instances of hemorrhage before or unrelated to radiosurgery were eliminated.

In addition to the primary papers, we meticulously scrutinized the references to identify potential cases meeting our specific inclusion criteria. Studies excluded from our consideration encompassed literature reviews, articles not available in full text, those reporting on unrelated diseases, pieces delving into diverse complications, and articles lacking comprehensive clinical information.

### Data extraction

Two investigators (AF and AM) independently extracted the following information from each included study: study characteristics (first author, year of publication, country, and study type) and participant characteristics. Any discrepancy in data extraction was resolved by discussion with the third author RV.

### Risk of bias assessment

The methodological quality of included studies was assessed independently by two reviewers using the Joanna Briggs Institute (JBI) critical appraisal tools: the JBI Checklist for Case Reports (8 criteria) for single-patient studies, and the JBI Checklist for Case Series (10 criteria) for multi-patient series. Disagreements were resolved by a third reviewer. Results are presented in Tables 1 and 2.

**Table 1:** JBI risk of bias—case reports

Study (Case Reports)	Demographics	History described	Condition at presentation	Diagnostic tests	Intervention described	Post-intervention outcome	Adverse events identified	Takeaway lessons
Miki et al. 2015 [14]	Y	Y	Y	Y	Y	Y	Y	Y
Thombre et al. 2019 [15]	Y	Y	Y	Y	Y	Y	Y	Y
Thompson et al. 1990 [16]	Y	Y	Y	U	Y	Y	U	Y
Karampelas et al. 2007 [17]	Y	Y	Y	Y	Y	Y	Y	Y
Dehdashti et al. 2009 [18]	Y	Y	Y	Y	Y	Y	U	Y

Y = Yes; N = No; U = Unclear

**Table 2:** JBI risk of bias-case series

Study (Case Series)	Inclusion criteria stated	Condition measured standardly	Valid measure used	Consecutive recruitment	Complete follow-up	Follow-up reported	Outcomes reported	Statistical analysis	Demographics reported	Clinical info reported
Bin-Alamer et al. 2024 [19]	Y	Y	Y	U	U	Y	Y	Y	Y	Y
Bin-Alamer et al. 2023 [20]	Y	Y	Y	U	U	Y	Y	Y	Y	Y
Murakami et al. 2011 [21]	Y	Y	Y	U	Y	Y	Y	N	Y	Y
Iwai et al. 2007 [22]	Y	Y	Y	U	Y	Y	Y	N	Y	Y
Iwai et al. 2003 [23]	Y	Y	Y	U	U	Y	Y	N	Y	Y

Consecutive recruitment and completeness of follow-up marked U where not explicitly reported. *N* = not performed (descriptive only)

### Statistical analysis

Due to heterogeneity among the included studies, quantitative meta-analysis was not performed. Instead, a qualitative narrative synthesis was conducted. For the purposes of this review, a “favorable outcome” was defined as any outcome reported by the original authors as clinical improvement, neurological stabilization, resolution or regression of hemorrhage on imaging, or absence of disease recurrence at last follow-up.

## Results

The initial literature search yielded 719 articles. After the removal of 173 duplicates, the title and abstract of 546 articles were screened and 531 articles were eliminated based on exclusion criteria. After this initial filter, 15 articles were assessed for eligibility, of which 5 were excluded because of lack of evidence of stereotactic radiosurgery. After screening, a total of 11 studies with 47 patients were included, all of whom developed intratumoral hemorrhage post-SRS. No cases included in the review had undergone other treatment modalities for the hemorrhage itself before radiosurgery. Thus, a total of 11 studies [14–24] encompassing 47 patients were eligible for review Fig. 1. The main characteristics of the included studies are presented in Table 3.

**Table 3:** Study characteristics of the included studies

S.NO	Author And Year of study	Type of study	Country	Number of Cases	Gender	Age	Risk Factors
1. 1	Bin-Alamer, Othman et al. 2024 [19]	Retrospective Case-series	Multi-instituitional	25	12 Males	62	6 patients with bleeding risk Factors, 2 having uncontrolled hypertension, 3 on antiplatelet medications, and 2 on anticoagulation therapy
					13 Females		
2. 2	Kensuk Murakami et al. 2011 [21]	Case -series	Japan	4	1 Male	37	N/A
					3 Female	39	N/A
						33	
						25	
3. 3	Shunichiro Miki et al. 2014 [14]	Case-report	Japan	1	1 Male	48	N/A
4. 4	Bhushan Thombre et al. 2019 [15]	Case-report	India	1	1 Male	63	Bleeding and clotting abnormalities
5. 5	B. Gregory Thompson et al. 1990 [16]	Case series	Sweden	1	1 Female	68	N/A
6. 6	I. Karampelas et al. 2007 [17]	Case report	Newyork	1	1 Male	53	Hypertension, coagulopathy and trauma
7. 7	Othman Bin-Alamer et al. 2023 [20]	Retrospective	Pennsylvania	5	3 Male	71	Hypertension
						71	Rivaroxaban for Atrial Fibrillation
						79	N/A
					2 Female	79	Apixaban for Atrial Fibrillation
						62	N/A
8. 8	Iwai et al. 2007 [22]	Case-series	Japan	3	3 Female	66	N/A
						71	
						56	
9. 9	Iwai et al. 2003 [23]	Retrospective Case- series	Pennsylvania	2	N/A	N/A	N/A
10. 10	Dehdashti et al. 2009 [18]	Case- report	Canada	1	1 Female	47	N/A
11	Moscovici et al. 2020 [25]	Case report	Israel/Australia	1	1 Female	67	Warfarin (mechanical AVR), hypertension, type 2 diabetes

### Patient demographics

**Fig. 2:**
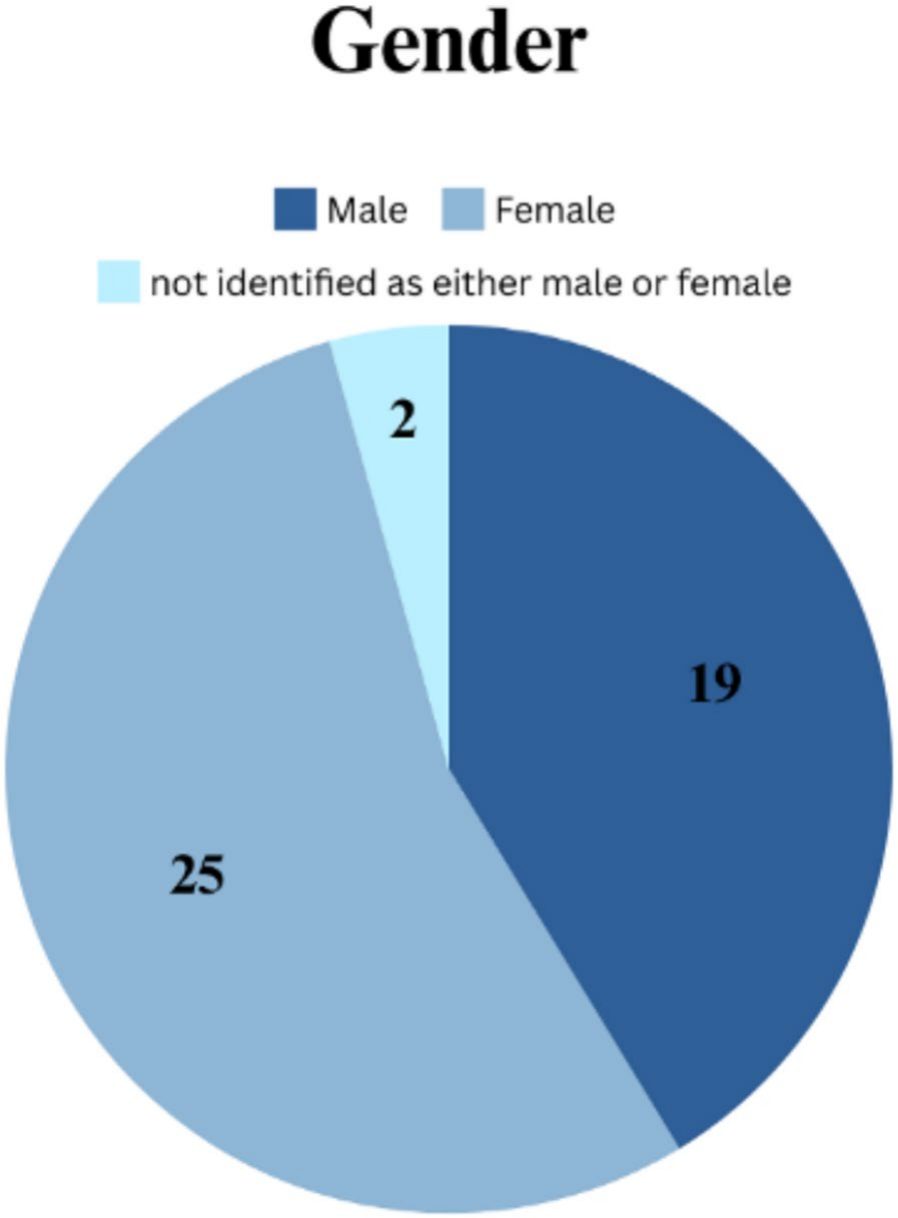
Proportion of gender distribution of the patients having intratumoral hemorrhage in vestibular schwannoma following the radiosurgery

The study encompassed a broad age range, with patients spanning from 25 to 79 years, yielding an average age of 52 years. Notably, the highest representation occurred among individuals in their sixth decade and those aged 60 years and above, shedding light on the prevalence of the condition in older demographics. In terms of gender distribution, 54% of the patients were female, while 4.3% were not distinctly identified as either male or female, as illustrated in Fig. 2.

### Presence of risk factors

**Fig. 3:**
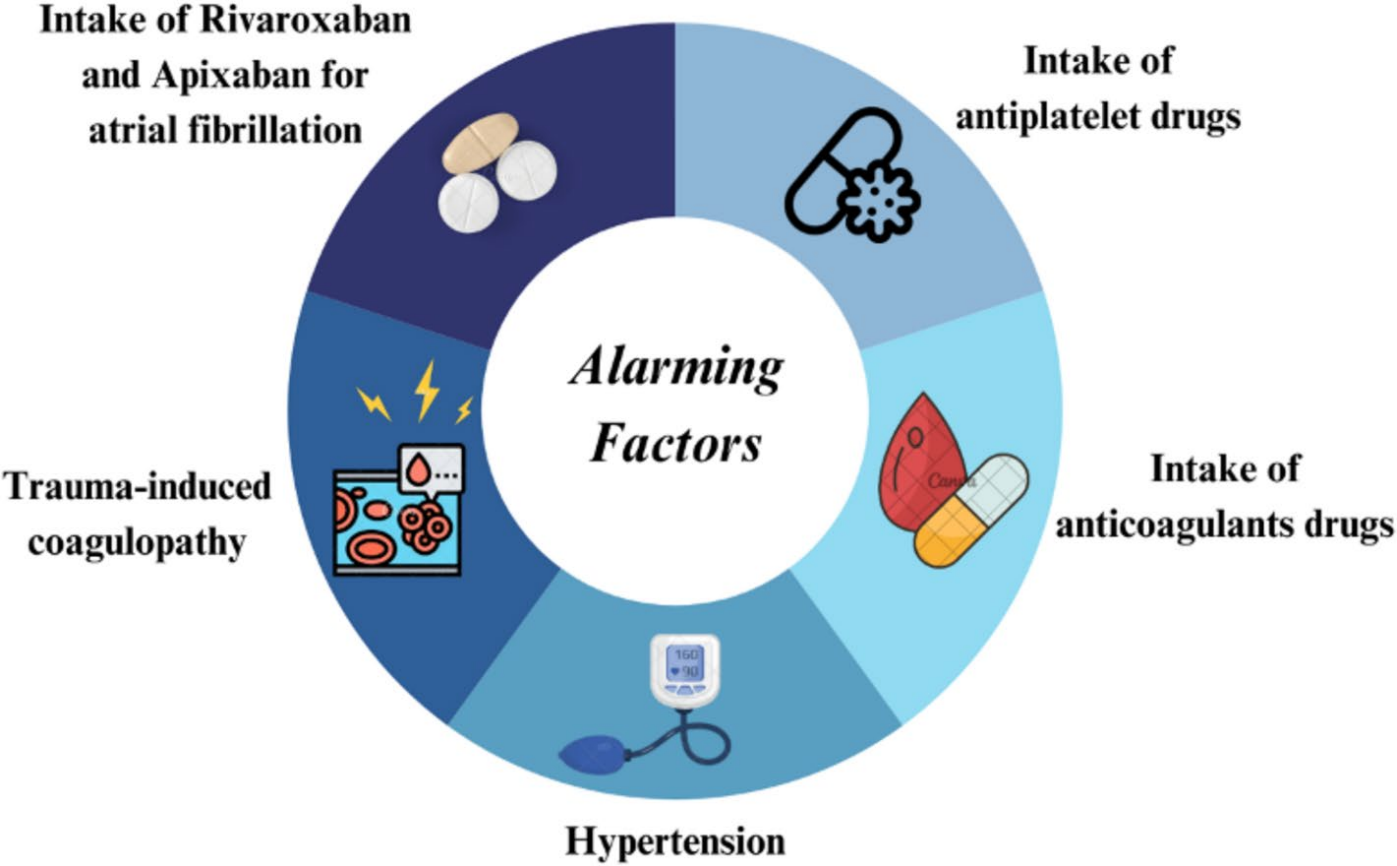
Risk factors of bleeding in patients considered for radiosurgery in the case of vestibular schwannoma

Among the 47 patients experiencing ITH post-stereotactic radiosurgery, a specific subgroup demonstrated discernible bleeding risk factors as depicted in Fig. 3. Six individuals displayed elevated susceptibility to bleeding, stemming from various factors (Table 3). Notably, two patients had uncontrolled hypertension, three were using antiplatelet medications, and two were undergoing anticoagulation therapy (Table 3). One case presented bleeding and clotting abnormalities, combined with hypertension and trauma-induced coagulopathy. Additionally, one patient had hypertension, and two were prescribed Rivaroxaban and Apixaban for atrial fibrillation (Table 3). This underscores the necessity of evaluating individual patient risk profiles when assessing the likelihood of ITH after SRS. It implies that pre-existing conditions and medications may play a substantial role in these events, highlighting the importance of targeted preventive strategies.

### Type of stereotactic radiosurgery and dosage of radiation

The predominant radiosurgical approach employed in the study was stereotactic radiosurgery (SRS), including Gamma Knife Radiosurgery (GKRS), which is one of the most commonly used techniques within the SRS spectrum. In the reviewed studies, the marginal radiation dose for SRS typically ranged between 12 Gy (Range: 12.0–12.3 Gy), with a maximum dose between 21.8 and 24.0 Gy, and an isodose line of 50−55%.

Within the SRS category, Gamma Knife Radiosurgery (GKRS) was frequently utilized, particularly for smaller tumors or patients with high surgical risks. The median marginal radiation dose for GKRS ranged between 11 Gy (Range: 10–12.5 Gy), and the maximum dose varied between 12 and Gy. The isodose lines used in different cases ranged from 46 to 50%.

In one unique case, Subtotal Microsurgical Resection followed by GKRS was used, with a prescribed dose of 13 Gy to the 46% isodose surface at the margins of the tumor, combined with a hemicraniectomy to treat large residual tumor. This shows the adaptability of SRS, including GKRS, in treating complex vestibular schwannomas.

### Interval for radiosurgery and presenting symptoms of ITH

Among the 47 patients, the timeframe between Radiosurgery and the onset of initial symptoms was documented for 34 individuals. Notably, five patients displayed symptoms at varying intervals: 2, 3, 6, 106, and 130 months post-radiosurgery. One patient experienced vestibular schwannoma (VS) enlargement and ITH 27 months after Gamma Knife Surgery (GKS). Another individual exhibited mass enlargement and irregular enhancement on T2-weighted imaging, indicating intratumoral hemorrhage, occurring 3 years and 6 months after Gamma Knife Radiosurgery (GKRS). Additionally, a patient-reported severe vertigo, heightened ataxia, and mild facial deviation ten days post-GKRS. Remarkably, 26 participants displayed a mean interval of 10 months between stereotactic radiosurgery (SRS) and the manifestation of intratumoral hemorrhage.

### Symptoms

The observed symptoms of ITH in VS following radiosurgery present a complex clinical picture presented in Tables 4 and 5. Trigeminal neuropathy is notably prevalent, affecting approximately 23.91% of the patients. Dysequilibrium, facial weakness, and facial sensory loss (V2, V3) collectively contribute to around 21.74%. Cerebellar ataxia, headache, and hearing decline or loss demonstrate prevalence rates of 17.39%, 13.04%, and 13.04%, respectively. The manifestation of facial pain, right facial dysesthesia, and right-side sensory loss in touch each represents around 4.35%. Various neurological deficits such as CN V–X palsy, hearing disturbance, facial palsy, hemifacial spasm, and severe facial motor paresis occur at lower frequencies. This heterogeneous symptomatology highlights the intricate nature of ITH in VS post-radiosurgery, necessitating individualized management approaches.

**Table 4:** Summary of symptoms, management and outcomes

S.NO	Author And Year of study	Patients	Symptoms	Management	Outcomes
	Bin-Alamer, Othman et al. 2024 [19]	12 Males 13 Females	Trigeminal neuropathy 11 Dysequilibrium 7 Facial weakness 5 Headache 4 Hearing decline 2 Hemifacial spasm 1	Initial observation with late surgical resection 2 (8%) Initial surgical intervention 2 (8%) Resection 1 VPS	Alive 23 Dead 2
	Kensuk Murakami et al. 2011 [21]	1 Male	N/A	Radical resection, Salvage microsurgery	Stable
		3 Female		Radical resection, Salvage microsurgery	Progression
			Right facial dysesthesia	Radical resection, Salvage microsurgery	Progression
			N/A	Radical resection, Salvage microsurgery	No Recurrence
1. 3	Shunichiro Miki et al. 2014 [14]	1 Male	Intratumoral infarction, bleeding	retrosigmoid approach, steroids and osmotherapy	Overall survival
	Bhushan Thombre et al. 2019 [15]	1 Male	Right-side hearing loss and tinnitus, sensory loss in touch and pain, intralesional hemorrhage, facial palsy	Oral steroids, surgical decompression	Overall survival
	B. Gregory Thompson et al. 1990 [16]	1 Female	Skew deviation, right-central facial weakness, mild right-sided hemiparesis and hemisensory loss, right-sided limb ataxia, dysmetria, and intention tremor	radiosurgery	Overall survival
	I.Karampelas et al. 2007 [17]	1 Male	Hearing loss and dizziness	radiosurgery	Overall survival
	Othman Bin-Alamer et al. 2023 [20]	3 Male	Left-side decreased hearing, giddiness, vomiting, and facial asymmetry	retrosigmoid suboccipital craniotomy	N/A
			Progressive hearing loss, severe facial motor paresis	retromastoid craniectomy with subtotal tumor resection	Overall survival
			Disequilibrium, facial pain, & facial weakness	observation	Symptoms improved; ITH dominant
		2 Female	Facial sensory loss (V2, V3) & disequilibrium	observation	Symptoms improved; ITH resolved
			Disequilibrium, headache, & hearing loss	observation	Symptoms improved; ITH resolved
	Iwai et al. 2007 [22]	3 Female	CN V–X palsy, cerebellar ataxia	radiosurgical procedure	Cerebellar ataxia improved
			Hearing disturbance cerebellar ataxia	radiosurgical procedure	CN VII palsy deteriorated, cerebellar ataxia improved but patient died of the expanding intratumoral hematoma
			Hearing disturbance, CN V, VII, and VIII palsy, cerebellar ataxia	radiosurgical procedure	CN V palsy and cerebellar ataxia improved
	Iwai et al. 2003 [23]	2	Cerebellar ataxia	radiosurgical procedure	CN V and VII palsy and cerebellar ataxia improved
			Facial pain	radiosurgical procedure	Good
	Dehdashti et al. 2009 [18]	1 Female	Headache, ataxia	Translabyrinthine approach and near total resection, VP Shun	Good
	Moscovici et al. 2020 [25]	1 Female	Disequilibrium, left facial weakness, hyperlacrimation, hemifacial spasm; then thunderclap headache, vomiting, diplopia, right abducens palsy	Warfarin reversal (Prothrombinex + Vitamin K), Nimodipine 21-day course, translabyrinthine surgical resection	Good recovery; dysequilibrium improved; left facial palsy unchanged (HB Grade 4)

**Table 5:** Percentage of different symptoms of ITH

Symptoms	Percentage
Trigeminal Neuropathy (including facial sensory loss V2/V3)	23.91%
Dysequilibrium	21.74%
Facial Dysfunction (including facial weakness, facial palsy, and facial asymmetry)	17.39%
Cerebellar Ataxia	17.39%
Headache	13.04%
Hearing Decline/Loss	13.04%
Facial Pain	6.52%
Right Facial Dysesthesia	4.35%
Right-Side Sensory Loss (Touch)	4.35%
Cranial Nerve (CN) Palsy (V–X)	4.35%
Hemifacial Spasm	2.17%
Severe Facial Motor Paresis	2.17%
Other Symptoms (e.g., skew deviation, dysmetria, intention tremor)	2.17%

### Treatment strategies

The diverse array of management strategies for ITH in VS post-radiosurgery underscores the complexity of managing such a complication. In the 47 patients, radiological monitoring (here referring to a “wait and scan” approach) emerged as the predominant intervention, constituting approximately 17.39% of cases (see Table 4). This strategy means that clinicians went to regular monitoring of patients with imaging to see the progression of the hemorrhage, rather than immediate intervention.

The intervention choice, accounting for 8.70%, also reflects a cautious approach in select cases where symptoms were stable, and immediate surgical intervention was not necessary. In contrast, radical resection coupled with salvage microsurgery was applied in 8.70% of patients, indicating the necessity of surgical intervention in cases where the hemorrhage did not stop, or where conservative management was not enough.

Less common procedures were also applied, including the retrosigmoid approach with steroids and osmotherapy, oral steroids with surgical decompression, retrosigmoid suboccipital craniotomy, and retromastoid craniectomy with subtotal tumor resection (each at 2.17%), as detailed in Table 4. These more invasive approaches were typically reserved for patients with more severe or deteriorating symptoms. Other interventions, such as initial observation with late surgical resection or translabyrinthine approaches (each at 2.17%), highlight the limited adoption of these techniques in clinical practice, as reflected in Table 4.

### Outcome

**Fig. 4:**
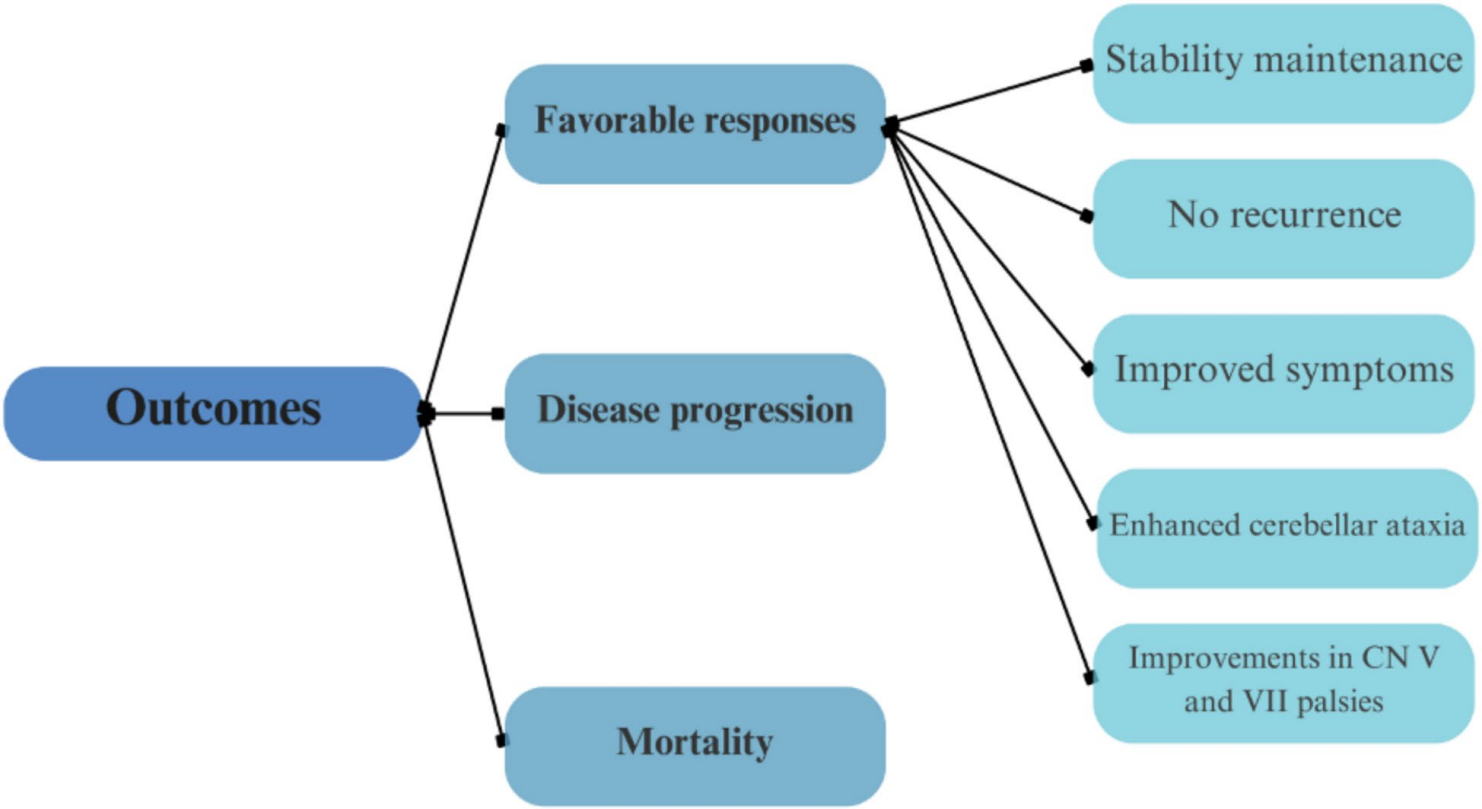
The outcomes that emerged after treating the intratumoral hemorrhage in patients with vestibular schwannoma who underwent radiosurgery

The outcomes that emerged after treating the intratumoral hemorrhage in patients with vestibular schwannoma who underwent radiosurgery are illustrated in Fig. 4. Among the 47 patients undergoing radiosurgery for ITH in a VS, approximately half demonstrated outcomes classified as favorable by the reporting authors, with 2.17% maintaining stability. Additionally, 4.35% exhibited no recurrence and 2.17% reported improved symptoms. These observations are descriptive summaries across heterogeneous reports and should not be interpreted as population-level estimates (Table 4). While 4.35% of patients, unfortunately, encounter disease progression, an equal percentage of 4.35% face mortality, revealing the complex challenges associated with managing ITH in VS post-radiosurgery.

## Discussion

Post-radiosurgery ITH in VS is considered to be a rare challenging entity [26–30]. Although uncommon, ITH can be associated with life-threatening clinical implications, hence highlighting the necessity to assess all possible outcomes for a better personalized treatment choice for VS patients. The objective of this systematic review is to comprehensively analyze the incidence, clinical implications, and characteristics ITH following radiosurgery, providing insights into its prevalence, risk factors, and potential impact on treatment outcomes.

This systematic review identified a heterogeneous distribution of patient demographics. Intriguingly, there was a notable clustering of cases among individuals in their sixth decade and older, suggesting a predilection for older age groups in the manifestation of this complication. Moreover, a slightly higher prevalence of ITH was observed among females, constituting 54% of documented cases. Consistent with our findings, a multi-institutional study conducted by *Bin-Alamer, Othman *et al*.* reported a median age of ITH onset at 62 years post-SRS in VS patients [19]. Although other existing studies primarily focused on associated other risk factors, older age and female gender should be further evaluated and considered as a potential determining characteristic in accordance to clinical setting.

This review is contextualized by three prior analyses. Niknafs et al. (2014) reviewed hemorrhagic VS and found spontaneous hemorrhage in 0.4–1% of cases, identifying tumor vascularity and cystic change as predisposing factors [26]. Carlson et al. (2017) reported clinically significant ITH in 1.6% of VS patients after radiosurgery and found tumor size and cystic change as risk factors [31]. The multi-institutional study by Bin-Alamer et al. (2024), which contributed 25 of the 47 patients in this review, corroborated these risk factors and additionally identified anticoagulant use as a contributor, with dysequilibrium as the most common presenting symptom [19]. Our findings are concordant with these reports. The additional case described by Moscovici et al. (2020), involving repeated ITH in an anticoagulated previously irradiated VS patient, further supports the compounding risk of anticoagulation in this setting [25].

Indeed, several factors, both inherent to tumor biology such as vascularity and necrosis, and external factors like hypertension, coagulopathy, and trauma, can collectively trigger hemorrhage within a tumor [32]. Larger tumor size, particularly those more than 3.0 cm, cystic development, hypointense T2-weighted MRI areas, hemosiderin deposition, and anticoagulant medication have all been associated to intratumoral hemorrhage (ITH) following radiosurgery in acoustic neuromas. Shahbazi et al. reported a 32× 25 × 26 mm tumor with a 20 × 15 × 5 mm hematoma, whereas Woo et al. described three patients with tumors ranging from 3.7 to 5.5 cm, all of whom required surgical intervention owing to hemorrhage-related problems such as brainstem compression and hydrocephalus. Around 10% of vestibular schwannomas (VS) have intracystic hemorrhage and cystic changes that increase the chance of numerous hemorrhagic episodes. Larger tumor volumes, particularly those larger than 3.0 cm, are associated with an increased risk of bleeding, emphasizing the necessity for prompt surgical intervention (Shahbazi et al., Woo et al*.* [17, 33, 34]) In addition to the incidents of ITH after SRS, it is critical to compare these data to the reported incidence of spontaneous bleeding in vestibular schwannomas without previous radiosurgery. The probability of hemorrhage occurrence in VS is low, occurring in 0.4% to 1% of cases. In contrast, post-SRS ITH appears to be affected by radiation dosage as well as patient-specific factors such tumor size and the use of anticoagulants. Current data shows that radiosurgery may increase the incidence of ITH, while direct comparative data between treated and untreated patients are missing [26, 35, 36]. Additional potential triggers might also include hypertension, excessive straining, pregnancy, any previous history of head trauma, or any history of illicit drug use [15, 37, 38]. These studies have also indicated that intratumoral hemorrhage within vestibular schwannomas can lead to acute clinical decline, with symptoms such as vertigo, gait imbalance, vomiting, and potential cranial nerve deficits [39]. As our review indicated, and amongst the 47 patients who experienced intratumoral hemorrhage following stereotactic radiosurgery, a distinct subset exhibited identifiable risk factors for bleeding. Hence, the presence of pre-existing conditions such as hypertension and the use of specific medications like Rivaroxaban and Apixaban for atrial fibrillation underscore the importance of assessing individual patient risk profiles to predict the likelihood of intratumoral hemorrhage post-SRS and to develop targeted preventive measures. Similarly, one study by *Bin-Alamer, Othman *et al.,* 2023,* focused on patients with VS who underwent GKRS and reported that factors such as tumor size, vascularity, and growth rate can contribute to intratumoral hemorrhage [19]. Additionally, the study identified bleeding risk factors in some patients who developed intratumoral hemorrhage post-GKRS, emphasizing the importance of patient-tailored management based on individual characteristics and presentation. It is evident that pre-existing medical conditions and medication regimens can significantly influence the occurrence of such complications. Therefore, tailored preventive strategies are essential to mitigate these risks effectively.

On the other hand, the type of radiosurgery performed, e.g. SRS or GKRS, as well as the dosage of radiation utilized, was assessed as potential risk factors for developing ITH post-VS radiotherapy. We observed a diverse array of modalities employed for treating these tumors, with notable variances in dosage parameters and treatment approaches. Indeed, various modalities, including Cyberknife® and Linac-based systems, were utilized for radiosurgical interventions [40]. These technologies offer different precision and radiation delivery mechanisms, potentially impacting treatment outcomes and associated risks. *Thombre, Bhushan *et al*.* noted the association of Gamma Knife radiosurgery and ITH with Gy ≤ 12 [15]. *Liu *et al*.* further noted the association of multiple microhemorrhages post GKRS, as well as microbleeding and hemosiderin disposition within VS [41]. *Thombre, Bhushan *et al. and *Carlson *et al. both agreed that microhemorrhage provides the inciting spark followed by a secondary factor that promotes further bleeding [15, 31]. We can indicate that, and while stereotactic radiosurgery remains a valuable treatment modality for VS, it is noted to be at increasingly higher risk of developing ITH. Hence, careful consideration of dosage parameters and treatment approach is essential to mitigate the risk of intratumoral hemorrhage, by focusing on refining treatment protocols, optimizing dose delivery, and elucidating the underlying mechanisms contributing to treatment-related complications, thereby enhancing patient outcomes and safety in the management of these tumors.

The interval between radiosurgery and the onset of symptoms was assessed to investigate it as an influencing factor for the clinical outcomes of ITH post-VS radiosurgery. Notably, while some patients exhibited symptoms relatively soon after treatment, others experienced delayed onset, with intervals extending beyond a year. Furthermore, the temporal relationship between radiosurgery and symptom onset varied across individuals. Trigeminal neuropathy emerges as the most prevalent symptom, affecting approximately 23.91% of patients, followed by dysequilibrium, facial weakness, and facial sensory loss (V2, V3) collectively contributing to around 21.74%. Other notable symptoms include cerebellar ataxia, headache, and hearing decline or loss, underscoring the complex clinical presentation of this condition and the need for personalized treatment strategies. *Karampelas, I *et al*. and *others noted that the majority of ITH cases are symptomatic, with headache, nausea, vomiting, facial pain, cerebellar ataxia, decreased level of consciousness and sudden cranial nerve deficit [17, 22, 36, 42]. Similarly, *Bin-Alamer, Othman *et al*.* noted that dysequilibrium was the most common presenting symptom of ITH, along with headache, facial weakness, and facial sensory dysfunction [20]. Our findings were also supported by other ITH reports that noted the predominance of acute hearing loss, facial paresis, trigeminal neuropathy, and headaches was the most common presentations of ITH, as depicted by *Mathkour, Mansour *et al. [43]*.* These symptoms mainly stem from the position of the tumor and the advancement of ITH, leading to heightened pressure within the skull and compression of nearby neurovascular structures such as the brainstem, facial nerve, and trigeminal nerve. Timely monitoring of patients for any new or worsening symptoms following radiosurgery for vestibular schwannoma is imperative to promptly address potential complications and optimize patient outcomes.

The management of intratumoral hemorrhage in vestibular schwannoma post-radiosurgery presents a multifaceted challenge, with various clinical manifestations and severity requiring individualized approaches. Studies have shown that the management of ITH post-SRS should be tailored to patient-specific conditions, considering factors such as the rapidity of progression, hemorrhage expansion, and overall patient status [19, 20]. Observation, urgent surgical intervention in select cases, and delayed resection due to hemorrhagic expansion or clinical deterioration constitute key management approaches. Patients with ITH may exhibit improvement or remain clinically stable following appropriate management, emphasizing the importance of timely intervention and monitoring. As *Bin-Alamer, Othman *et al*.* indicated, the treatment of choice mainly depends on the acuteness of presentation, size of the hemorrhage, presence of expansion, and degree of impingement on surrounding neurovascular structures [20]. *Thombre, Bhushan *et al*.* advised to note that tumor growth coupled with neurological decline, as it can result from intratumoral hemorrhage post-GKRS, potentially necessitating swift surgical decompression to relieve compression-related symptoms [15, 24, 44]. Additionally, it is crucial to recognize that the morbidity and mortality rates following VS surgery are elevated in cases involving hemorrhage compared to those without, emphasizing the need for cautious management strategies.

The most common outcome observed in this systematic review was favorable response, followed by stability maintenance and no recurrence. Additionally, symptom improvement related to intratumoral hemorrhage and enhancements in cerebellar ataxia or cranial nerve V and VII palsies were frequently reported. However, disease progression and mortality were also commonly encountered, emphasizing the complex challenges in managing intratumoral hemorrhage in vestibular schwannoma post-radiosurgery. The complexities observed in our review necessitate a multidisciplinary approach to the management of intratumoral hemorrhage in vestibular schwannomas/acoustic neuromas. Close collaboration between neurosurgeons, radiation oncologists, and other relevant specialists is crucial for optimizing treatment outcomes and minimizing adverse events. Additionally, further research is warranted to elucidate the factors influencing patient responses to radiosurgery and to refine treatment protocols accordingly.

## Limitations

This review has several important limitations. First, all included studies are case reports or small case series with inherently high risk of bias. Second, quantitative meta-analysis was not possible given study heterogeneity. Third, outcome definitions were not standardized across studies. Fourth, no comparator group of untreated patients was available, precluding causal conclusions about SRS-attributable hemorrhage risk. Future prospective registries with standardized outcome reporting are needed.

The overall certainty of evidence for all outcomes in this review is rated as Very Low under the GRADE framework. This reflects exclusive reliance on case reports and series without comparator groups, high risk of selection bias, absence of standardized outcome definitions, inconsistent follow-up duration, and a small total sample (*n* = 47). All reported frequencies and outcome rates represent descriptive summaries only and should not be interpreted as epidemiological estimates.

## Conclusion

In conclusion, the management of intratumoral hemorrhage in vestibular schwannoma post-radiosurgery presents both opportunities and challenges in clinical practice. This systematic review sheds light on the diverse spectrum of patient outcomes, reflecting the complexity of this condition and the multifaceted nature of its management. While radiosurgery demonstrates considerable potential in managing intratumoral hemorrhage, with disease progression and mortality in a subset of patients highlighting the need for vigilant monitoring and multidisciplinary intervention.

The variability in patient outcomes highlights the importance of individualized treatment approaches tailored to specific patient characteristics and clinical presentations. Factors such as tumor size, growth rate, neurological deficits, and patient preferences should inform treatment decisions, with close collaboration between specialists essential for optimizing outcomes and minimizing complications.

A direct comparison of hemorrhage occurrences among patients undergoing SRS against those who do not reveals that, while spontaneous hemorrhage is uncommon, radiosurgery may raise the risk in people with certain risk factors such as big tumor size and anticoagulant use. Further studies should directly compare these populations in order to better understand the additional risk caused by radiosurgery.

## Supplementary Information

Supplementary Material 1.

## Data Availability

No datasets were generated or analysed during the current study.
