# Supplementary material for: Intratumoral hemorrhage in vestibular schwannomas post-radiosurgery: a systematic review
Source: Chin Neurosurg J. 2026 Jun 26;12:18. doi: 10.1186/s41016-026-00439-5 (PMC13308183; doi:10.1186/s41016-026-00439-5)
Supplement: Supplementary file 1 — Supplementary Material 1. [file 41016_2026_439_MOESM1_ESM.docx]

|  |  | **Supplementary Table 1: Detailed Search Strategy** |  |
| --- | --- | --- | --- |
| **Database** | **S.NO.** | **Search String** | **Search Results** |
|  | 1 | " intratumoral hemorrhage” | 33,900 |
| **Google Scholar** | 2 | "acoustic neuroma" OR "vestibular schwannoma" | 17,300 |
|  | 3 | “Stereotactic radiosurgery” OR "gamma knife radiosurgery" | 17,900 |
|  | **4** | **1-3** | **451** |
| **PubMed** | 5 | " ("intratumor"[All Fields] OR "intratumoral"[All Fields] OR "intratumorally"[All Fields] OR "intratumorous"[All Fields] OR "intratumour"[All Fields] OR "intratumoural"[All Fields] OR "intratumourally"[All Fields]) AND ("blood"[MeSH Subheading] OR "blood"[All Fields] OR "blood"[MeSH Terms] OR "bloods"[All Fields] OR "haematology"[All Fields] OR "hematology"[MeSH Terms] OR "hematology"[All Fields] OR "haematoma"[All Fields] OR "hematoma"[MeSH Terms] OR "hematoma"[All Fields] OR "haemorrhage"[All Fields] OR "hemorrhage"[MeSH Terms] OR "hemorrhage"[All Fields] OR "haemorrhages"[All Fields] OR "hemorrhages"[All Fields] OR "haemorrhagic"[All Fields] OR "haemorrhaging"[All Fields] OR "hematologies"[All Fields] OR "haematomas"[All Fields] OR "hematomas"[All Fields] OR "hematoma s"[All Fields] OR "hematomae"[All Fields] OR "hemorrhaged"[All Fields] OR "hemorrhagic"[All Fields] OR "hemorrhagical"[All Fields] OR "hemorrhaging"[All Fields])” | 10,225 |
|  | 6 | "neuroma, acoustic"[MeSH Terms] OR ("neuroma"[All Fields] AND "acoustic"[All Fields]) OR "acoustic neuroma"[All Fields] OR ("acoustic"[All Fields] AND "neuroma"[All Fields]) OR ("neuroma, acoustic"[MeSH Terms] OR ("neuroma"[All Fields] AND "acoustic"[All Fields]) OR "acoustic neuroma"[All Fields] OR ("vestibular"[All Fields] AND "schwannoma"[All Fields]) OR "vestibular schwannoma"[All Fields]) | 11,330 |
|  | 7 | ("stereotactic"[All Fields] OR "stereotactical"[All Fields] OR "stereotactically"[All Fields] OR "stereotactics"[All Fields]) AND ("radiosurgery"[MeSH Terms] OR "radiosurgery"[All Fields] OR ("gamma"[All Fields] AND "knife"[All Fields] AND "radiosurgery"[All Fields]) OR "gamma knife radiosurgery"[All Fields]) | 17,449 |
|  | **8** | **5-7** | **167** |
| **Cochrane Library** | **9** | **4, 8** | **101** |
